# The effects of DENV serotype competition and co-infection on viral kinetics in *Wolbachia*-infected and uninfected *Aedes aegypti* mosquitoes

**DOI:** 10.1186/s13071-021-04816-0

**Published:** 2021-06-09

**Authors:** M. Novelo, M. D. Audsley, E. A. McGraw

**Affiliations:** 1 School of Biological Sciences, Monash University, Melbourne, VIC 3800 Australia; 2 Center for Infectious Disease Dynamics, Department of Entomology, The Huck Institutes of the Life Sciences, The Pennsylvania State University, University Park, PA 16802 USA

**Keywords:** Dengue, Co-infection, *Aedes aegypti*, Serotype, *Wolbachia*, Infection dynamics

## Abstract

**Background:**

The *Aedes aegypti* mosquito is responsible for the transmission of several medically important arthropod-borne viruses, including multiple serotypes of dengue virus (DENV-1, -2, -3, and -4). Competition within the mosquito between DENV serotypes can affect viral infection dynamics, modulating the transmission potential of the pathogen. Vector control remains the main method for limiting dengue fever. The insect endosymbiont *Wolbachia pipientis* is currently being trialed in field releases globally as a means of biological control because it reduces virus replication inside the mosquito. It is not clear how co-infection between DENV serotypes in the same mosquito might alter the pathogen-blocking phenotype elicited by *Wolbachia* in *Ae. aegypti.*

**Methods:**

Five- to 7-day-old female *Ae. aegypti* from two lines, namely, with (*w*Mel) and without *Wolbachia* infection (WT), were fed virus-laden blood through an artificial membrane with either a mix of DENV-2 and DENV-3 or the same DENV serotypes singly. Mosquitoes were subsequently incubated inside environmental chambers and collected on the following days post-infection: 3, 4, 5, 7, 8, 9, 11, 12, and 13. Midgut, carcass, and salivary glands were collected from each mosquito at each timepoint and individually analyzed to determine the percentage of DENV infection and viral RNA load via RT-qPCR.

**Results:**

We saw that for WT mosquitoes DENV-3 grew to higher viral RNA loads across multiple tissues when co-infected with DENV-2 than when it was in a mono-infection. Additionally, we saw a strong pathogen-blocking phenotype in *w*Mel mosquitoes independent of co-infection status.

**Conclusion:**

In this study, we demonstrated that the *w*Mel mosquito line is capable of blocking DENV serotype co-infection in a systemic way across the mosquito body. Moreover, we showed that for WT mosquitoes, serotype co-infection can affect infection frequency in a tissue- and time-specific manner and that both viruses have the potential of being transmitted simultaneously. Our findings suggest that the long-term efficacy of *Wolbachia* pathogen blocking is not compromised by arthropod-borne virus co-infection.

**Graphic Abstract:**

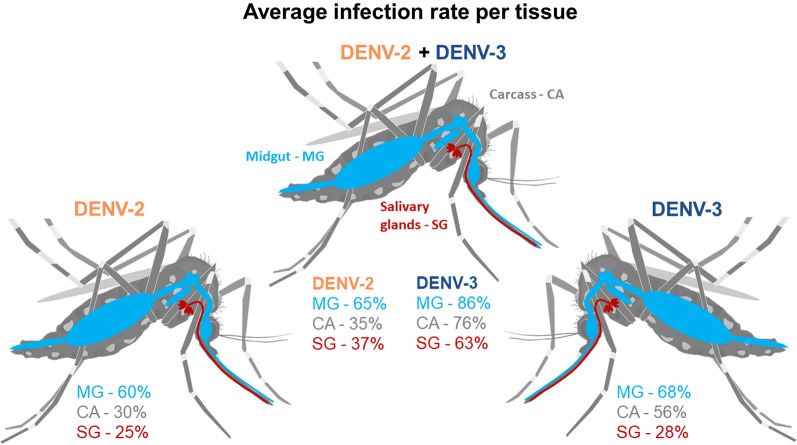

**Supplementary Information:**

The online version contains supplementary material available at 10.1186/s13071-021-04816-0.

## Background

Dengue viruses (DENVs) are medically important arthropod-borne viruses (arboviruses) responsible for up to 300 million cases of dengue fever a year, and they can be caused by any of the four related but antigenically distinct DENV serotypes (DENV-1 to DENV-4) [1]. In regions with endemic transmission of all four serotypes of DENV, varying predominance of certain serotypes has been observed between seasons [2]. Differences in the DENV infection kinetics and transmission potential are influenced by the genetic diversity of the four different DENV serotypes. The overall genome sequence-level differences between serotypes are estimated at 20–30% [3, 4].

Substantial evidence indicates that variation in the DENV genome of serotypes and strains can have epidemiological significance by altering the extrinsic incubation period (EIP) [5–7], defined as the time it takes for the pathogen to be transmitted by the vector [8], and therefore has a powerful effect on the scale and speed of epidemics. DENV-2 strains from the American and Southeast Asian genotypes differ in their EIP lengths, with the Southeast Asian genotypes having shorter EIPs. This shorter EIP was thought, in part, to explain the displacement of the American DENV strains in South America by the Asian lineage [9]. Additionally, different DENV serotypes exhibit various degrees of infectivity across the same mosquito populations [10–14]. Moreover, oral susceptibility to DENV-1 was shown to be up to four times higher than that of DENV-3 in *Ae. aegypti* from Senegal, with DENV-1 having higher infection and dissemination rates [15]. Finally, systematic analyses of DENV replication kinetics of all four DENV serotypes found significant differences in the infection rate and EIP between serotypes [13, 14].

Competition between DENV strains and serotypes can also affect viral population dynamics within the vector, thus modulating the transmission potential [16, 17]. This happens in nature when mosquitoes take multiple blood meals from several different hosts that are each infected with a different or multiple DENV serotypes [18]. In controlled laboratory experiments, using field-derived mosquito populations, there were no differences in dissemination and transmission rates between DENV-1 and DENV-4 mono-infections in *Ae. aegypti*, but during co-infection, DENV-4 had a much higher dissemination rate, leading to the exclusive presence of DENV-4 in the saliva for this particular experimental mosquito population [19]. Furthermore, differential replication between DENV-2 and DENV-3 has been shown, with DENV-2 exhibiting a much higher replication efficiency both in vitro and in vivo during co-infection [20]. Additionally, the effect of co-infection with different families of arboviruses on vector competence has just been recently studied. For example, mosquitoes exposed to double or triple infections with chikungunya virus (CHIKV; *Togaviridae*), Zika virus (ZIKV; *Flaviviridae*), and DENV (*Flaviviridae*) were capable of transmitting all pathogens concurrently, without noticeable changes to mosquito infection and dissemination rates [21]. Co-infection studies may shed light on the outcome of competitive processes in field mosquitoes, even if rare, but also importantly allow for direct comparisons of the transmissibility between viruses.

Given the ease of rearing *Ae. aegypti* in the laboratory, vector competence experiments are an important tool with which to study the effect and interaction between DENV and mosquito genotypes in the transmission potential of the virus. However, one of the main issues with artificial vector competence experiments is that there is too much heterogeneity between experiments that results from environmental variation and its interaction with genetic variation in both the mosquitoes and viruses [22]. Individual vector competence experiments using single DENV serotypes or strains often give varying results in both infection and transmission rates, making pairwise comparisons difficult to interpret. Moreover, limited data are available on co-infections with different serotypes, with some experiments suggesting competitive disadvantage or superinfection interference between DENV serotypes [23]. Additionally, it is not clear how these viral dynamics and interactions may be altered in the presence of *Wolbachia* infection, which is currently being trialed in global releases as a means of reducing virus transmission to humans [24] and that is known to reduce viral replication in serotype-specific ways [25–27]. To the best of our knowledge, only one study has looked at the effects of arboviral co-infection in *Wolbachia-*infected mosquitoes using ZIKV and DENV [28].

To assess the effect of DENV serotype co-infection on transmissibility and any corresponding interactions with *Wolbachia* infection, we used two interdependent approaches. First, we challenged two *Ae. aegypti* mosquito lines that were either *Wolbachia* infected (*w*Mel) or *Wolbachia* uninfected (WT) in both mono- and co-infection vector competence experiments with DENV-2 and DENV-3. We collected midgut, carcass, and salivary glands at nine time points post-infection. We then used the infection rate and viral RNA load data to assess the effects of competing serotypes and evaluate their performance in individual mosquitoes and between *w*Mel and WT lines. Second, we determined whether serotype co-infection altered viral infection dynamics relative to the mono-infected state by comparing viral RNA load and infection rate between the two vector competence experiments.

## Methods

### Mosquito lines and rearing

The mosquito lines used for this work have been described previously [29, 30]. The *w*Mel line was collected from the *Wolbachia* release zone in Cairns, Australia, as part of the Eliminate Dengue Program, whereas the WT line, naturally free from *Wolbachia*, was collected outside the *Wolbachia* release zone. Both lines were identified morphologically and with genetic markers as well as screened for the presence/absence of pathogens before being used in our study [31]. WT and *w*Mel larvae were fed TetraMin^®^ fish food (Melle, Germany), and adults were maintained on 10% sucrose. All mosquitoes were reared in a controlled environment at 26 °C, 75% relative humidity, and a 12-h light/dark cycle.

### Virus culture and titration

The DENV serotypes/strains used for this experiment are listed in Table 1. The virus was propagated in cell culture, as described previously [31]. Briefly, *Ae. albopictus* C6/36 cells were grown at 26 °C in RPMI 1640 medium (Invitrogen, Carlsbad, CA) supplemented with 10% fetal bovine serum (FBS), 1 × GlutaMAX (Invitrogen), and HEPES buffer. Cells were first allowed to form monolayers of around 60–80% confluence in T-175 flasks (Sigma-Aldrich, St. Louis, MO), and then they were inoculated with DENV and maintained in RPMI medium supplemented with 2% FBS. After 7 days post-inoculation, live virus was harvested, titrated via absolute quantification PCR (qPCR) and plaque-forming unit assay (as per below), and adjusted to a final concentration of ~ 4 × 10^5^ plaque-forming units (PFU)/ml for both serotypes (Table 2) prior to mixing with blood. Live virus was used for all vector competence experiments.

**Table 1:** Dengue serotypes and strains used

Serotype	Strain	Passage	GenBank accession number	Place of origin	Collection date
DENV-2	ET300	11	EF440433.1	East Timor	2000
DENV-3	Cairns 2008	9	JN406515.1	Australia	2008

**Table 2:** Viral titers

Serotype	Undiluted RT-qPCR titer of infected supernatant (copies/ml)	Final plaque assay titer for the blood meal (PFU/ml)	Average ratio of viral RNA copies/ml relative to PFU/ml
DENV-2	4 × 10^7^	~ 3 × 10^5^	277.1
DENV-3	8 × 10^8^	~ 4 × 10^5^	1382.1

Prior to the above steps, we isolated the viruses at different time points from C6/36 cells and assessed their viral RNA loads by qPCR and plaque assay to select the most appropriate day to harvest virus for the vector competence experiments. These pilot experiments also revealed the relationship between viral RNA load estimates by qPCR and live virus estimates by plaque assay. In general, we saw that higher viral RNA loads correlated with higher plaque assay titers (Additional file 1: Figure 1), with viral RNA loads ranging from ~ 10- to 2000-fold higher than live virus titers, as expected [32, 33]. The average ratio of viral RNA copies relative to PFU/ml was larger for DENV-3 (1300-fold) than for DENV-2 (270-fold), indicating that RT-qPCR was more sensitive for DENV-3. This effect was consistent across the two tested collection time points.

### Mosquito infections

The methods for mosquito oral infections have been described previously [14, 29]. Briefly, prior to oral DENV infections, 6- to 7-day-old adult female mosquitoes were sorted and starved for 24 h. For mono-infections, a 1:1 mix of virus culture and defibrinated sheep blood was prepared. For co-infections, 1 ml of each DENV serotype was combined, and from that blend, 1 ml was combined with 1 ml of defibrinated sheep blood. Glass feeders with double chambers were covered with pig intestine, and water heated to 37 °C was circulated in the outer chamber of the feeders. The mosquitoes were allowed to feed for ~ 2 h. Immediately after blood feeding, mosquitoes were knocked down and sorted on ice. Unfed females were discarded. The remaining mosquitoes were returned to 70-ml plastic cups and maintained on 10% sucrose. At days post-infection (DPI) 3, 4, 5, 7, 8, 9, 11, 12, and 13, mosquitoes were anesthetized and dissected in sterile phosphate-buffered saline (PBS). We collected midgut, carcass, and salivary glands from individual mosquitoes. For our vector competence experiments, the mosquito carcass was the collection of tissues that remained after dissecting the midgut and salivary glands. We used the carcass as a proxy for viral dissemination from the mosquito midgut. All tissue collections were conducted on live mosquitoes. Individual tissues were collected in 1.5-ml microcentrifuge tubes (Sarstedt, Nümbrecht, Germany) containing 200 µl of TRIzol reagent (Invitrogen) and 2-mm glass beads. Samples were homogenized and frozen at − 80 °C until RNA extraction.

### DENV absolute quantification via RT-qPCR

The RT-qPCR mixture contained 2.5 μl of LC480 master mix (Roche); 4.25 μl of PCR-grade water; 0.25 μl of 10 μM forward (F) primer, reverse (R) primer, and probe (P) specific to each DENV serotype; and 2 μl of RNA. Reactions were run in the LightCycler 480 instrument (Roche), and the thermal cycling conditions were 95 °C for 3 min, 30 cycles of 95 °C for 30 s, 50 °C for 1 min, and 68 °C for 1 min, finalizing with 68 °C for 5 min. Standard curves were generated by triplicate on each plate by analyzing 100 to 10^7^ copies/reaction of DENV fragment copies with a limit of detection set at 100 copies. DENV genome copies were extrapolated from the standard curve as DENV copies per tissue. The standards for both serotypes were generated using a ~ 100-bp conserved region of the NS5 protein of the DENV genome. The detection threshold for both DENV-2 and DENV-3 was set at ~ 35 Ct. The primers and probes used for the detection of specific DENV serotypes were as follows:

#### DENV-2-ET300

F primer (primer 9,873,681; TCCATACACGCCACACATGAG).

R primer (primer 98,736,818; GGGATTTCCTCCCATGATTC).

Probe–FAM (probe 98,084,286; 56-fam/AGGGTGTGGATTCGAGAAAACCCATGG/3BHQ_1).

#### DENV-3-Cairns08/09

F primer (primer 98,644,632; TTTCTGCTCCCACCACTTTC).

R primer (primer 98,451,787; CCATCCYGCTCCCTGAGA).

Probe–LC640 (probe9845178; 5LtC640N/AAGAAAGTTGGTAGTCCCCTGCAGACCTCA/3IAbRQSp).

### Statistical analysis

All statistical analysis used R v3.6.0 (http://www.r-project.org/). For the analysis of the infection frequency, a one-way ANOVA was fitted, and Tukey for contrasts was used for post hoc comparisons. Viral RNA load analysis was carried out using Kruskal–Wallis by rank test for the non-parametric data. All DENV RNA loads were reported on a log10 scale given the value range.

## Results

In this study, we challenged both WT and *w*Mel *Ae. aegypti* mosquitoes with two serotypes in the co- and mono-infected states. Specifically, we investigated (1) the relative infection dynamics of two DENV serotypes inside the mosquito by competing them directly, and (2) how individual serotypes behave when they are in a co-infection relative to mono-infection. Mosquitoes were fed blood with DENV-2 and DENV-3, both separately and together. In the case of mono-infections, a 1:1 mix of each DENV serotype and blood was used. For co-infections, the two serotypes were first combined, and from that blend, a 1:1 mix of blood and the two serotypes was used. Overall, the viral titers for both experiments were equivalent. We used RT-qPCR with serotype-specific TaqMan probes to quantify DENV RNA load in midgut, carcass, and salivary gland tissues to assess dissemination and infectiousness at nine time points post-infection.

### DENV serotype competition in co-infection

**Fig. 1:**
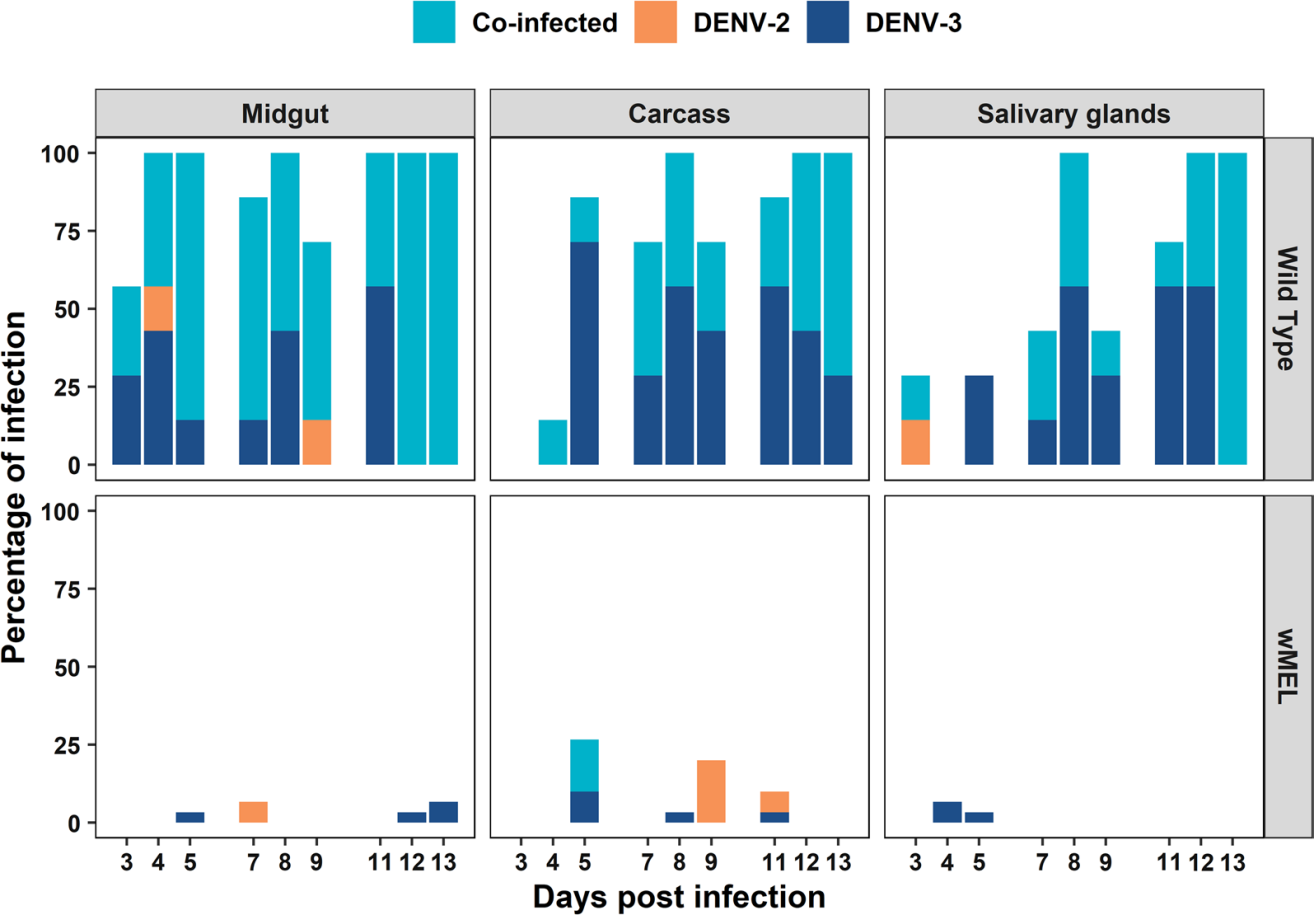
Mosquito susceptibility to DENV in co-infections by DPI, tissue, and line. *Ae. aegypti* were orally challenged with DENV-2 (orange) and DENV-3 (navy) simultaneously, whereby mosquitoes were fed a blood meal containing both viruses at ~ 3 × 10^5^ DENV genome copies/ml. Mosquito tissues (midgut, carcass, and salivary glands) were collected at nine time points post-infection. DENV RNA load was determined via RT-qPCR using serotype-specific probes. Each bar represents the proportion of mosquitoes positive for either (orange or navy) or both (blue) serotypes for each day post-infection. Total number of mosquitoes screened per day was *n* = 7 for the wild-type line and *n* = 30 for *w*Mel

By competing the serotypes in same mosquitoes, we were able to powerfully compare their performance, controlling some of the substantial variation that can occur across vector competence experiments. Infection rates in the WT mosquito line indicate that DENV-3 was a better competitor than DENV-2 but that the magnitude of this difference changed depending on the tissue and DPI. In the co-infection experiments, we classified the infection rates as uninfected (no viral RNA load detectable for either serotype), only DENV-2 infected (DENV-2/alone), only DENV-3 infected (DENV-3/alone), or co-infected (both serotypes detected). We saw significant variation in the percentage of infected WT mosquitoes between serotypes (df = 1, *F* = 22.2, *P* < 0.0001). In the midgut, most mosquitoes were co-infected at all DPIs (infection rate: 25–100%). Mosquitoes were DENV-3/alone at six time points with an infection rate between 10 and 50%, and DENV-2/alone at DPI 4 and 9 with an infection rate of ~ 15% (Fig. 1, midgut).

In the carcass, most WT mosquitoes were either co-infected or DENV-3/alone, but we saw no DENV-2/alone mosquitoes at any time point (Fig. 1, carcass). For the salivary glands, most WT mosquitoes were similarly either co-infected or DENV-3/alone, and there was only one time point at which we observed DENV-2/alone mosquitoes, with an infection frequency of ~ 10% at DPI 3 (Fig. 1, salivary glands). For *w*Mel mosquitoes, no pairwise comparison between serotypes was possible, as there were too few infected mosquitoes to perform statistical analysis, due to the action of *Wolbachia*-mediated pathogen blocking. Overall, our results indicate that DENV-3 is better at replicating and disseminating in mosquitoes, regardless of tissue or time point, than DENV-2.

**Fig. 2:**
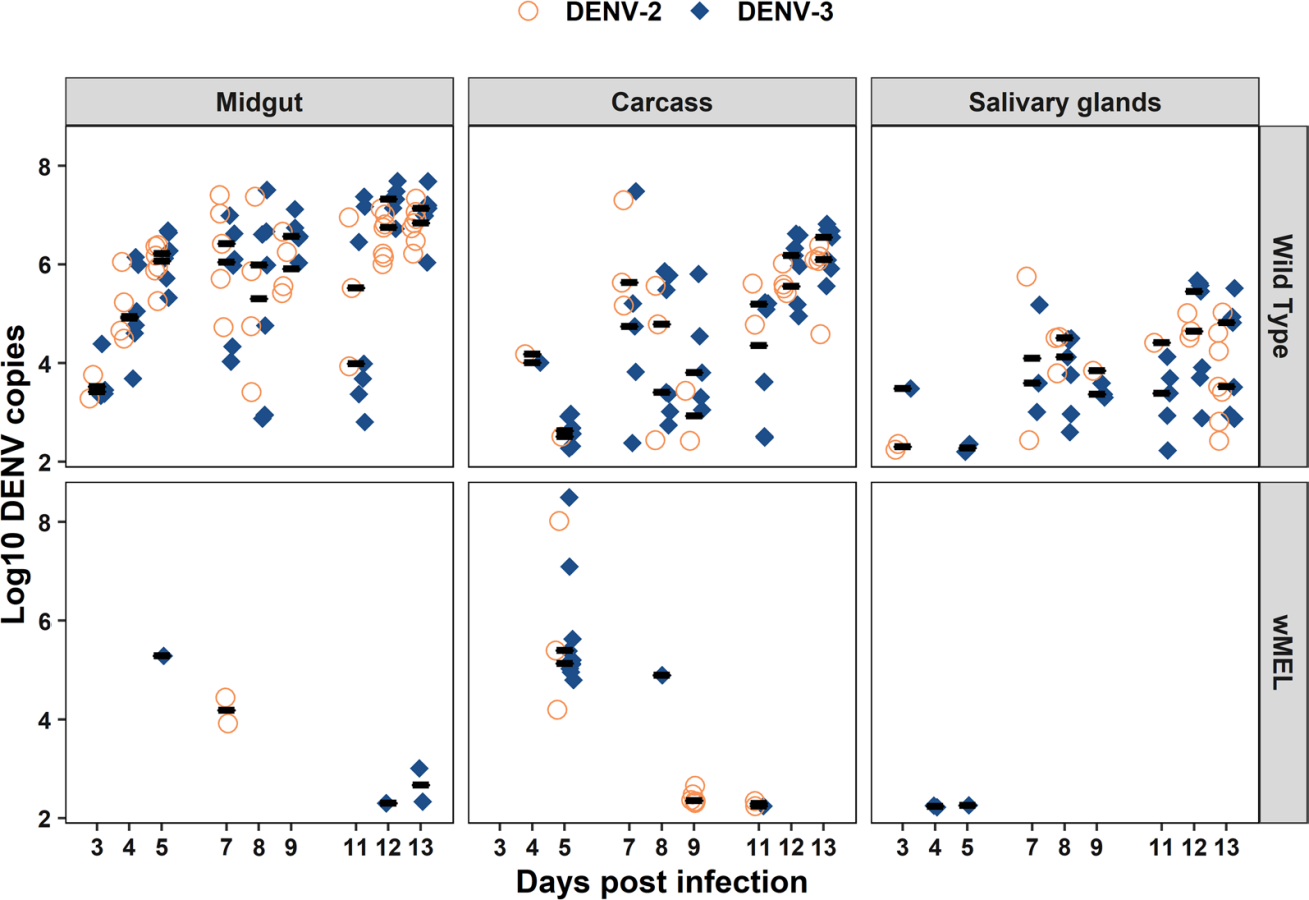
DENV load in co-infection by DPI, tissue, and mosquito line. *Ae. aegypti* were orally challenged with DENV-2 (orange circles) and DENV-3 (navy diamonds) simultaneously, and both viruses were fed to mosquitoes at ~ 3 × 10^5^ DENV genome copies/ml. Mosquito tissues (midgut, carcass, and salivary glands) were collected at nine days post-infection, and DENV RNA load was determined via RT-qPCR using serotype-specific probes. Mosquitoes with undetectable viral RNA load are not represented in this graph. Black bars represent treatment medians. Each symbol represents a single mosquito sample. Total number of mosquitoes screened per day was *n* = 7 for the wild-type line and *n* = 30 for *w*Mel

We also compared viral RNA loads between serotypes across tissues and DPI. Overall, we saw no significant differences between DENV-2 and DENV-3 (Fig. 2, *χ*^2^ = 1.66, *P* = 0.19, df = 1). The viral RNA load for both serotypes in the WT mosquitoes ranged from ~ 10^3^ to ~ 10^8^ in the midgut, ~ 10^2^ to ~ 10^7^ in the carcass, and ~ 10^2^ to ~ 10^6^ log10 DENV copies per tissue in the salivary glands. In contrast, the viral RNA load in the *w*Mel mosquitoes was diminished and only observed in 10 time points across all tissues, ranging from ~ 10^2^ to ~ 10^5^ in the midgut, ~ 10^2^ to ~ 10^8^ in the carcass, and ~ 10^2^ log10 DENV copies per tissue in the salivary glands. Additionally, in the *w*Mel mosquitoes, viral RNA loads from both serotypes were observed at only one time point (Fig. 1, *w*Mel, carcass, DPI 5).

**Fig. 3:**
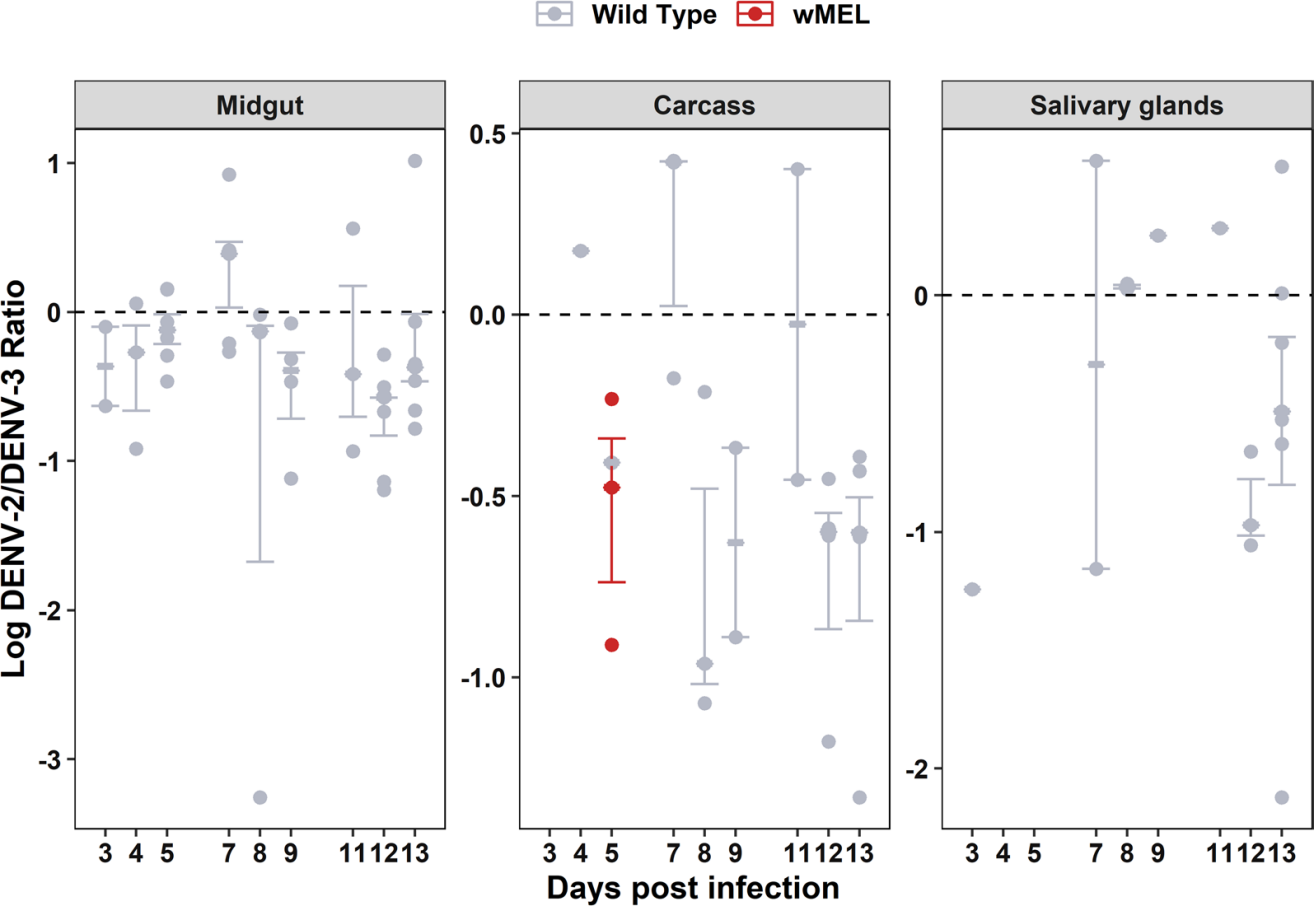
Co-infection serotype ratio by DPI and tissue. Graphs depict the log10 DENV-2/DENV-3 ratio for all wild-type (grey) and *w*Mel (red) samples found to be positive for infection with both viruses via RT-qPCR for each tissue. Ratios greater than zero indicate that DENV-2 levels were higher than DENV-3 levels for that sample. Color lines represent mean DENV-2/DENV-3 ratio and the standard error estimate of the mean ratio

Viral RNA loads from both serotypes were expressed as DENV-2/DENV-3 ratio (Fig. 3). Although for all tissues there was a general trend of co-infected samples having higher levels of DENV-3 than DENV-2, they were not significantly different from one another. For the *w*Mel mosquitoes, only DPI 5 had sufficient data with which to calculate a ratio due to the action of *Wolbachia* pathogen blocking; in this case, DENV-3 appeared higher than DENV-2, but with so few data points, statistical comparisons were not possible.

### Co-infection alters infection dynamics of DENV serotypes in WT mosquitoes

**Fig. 4:**
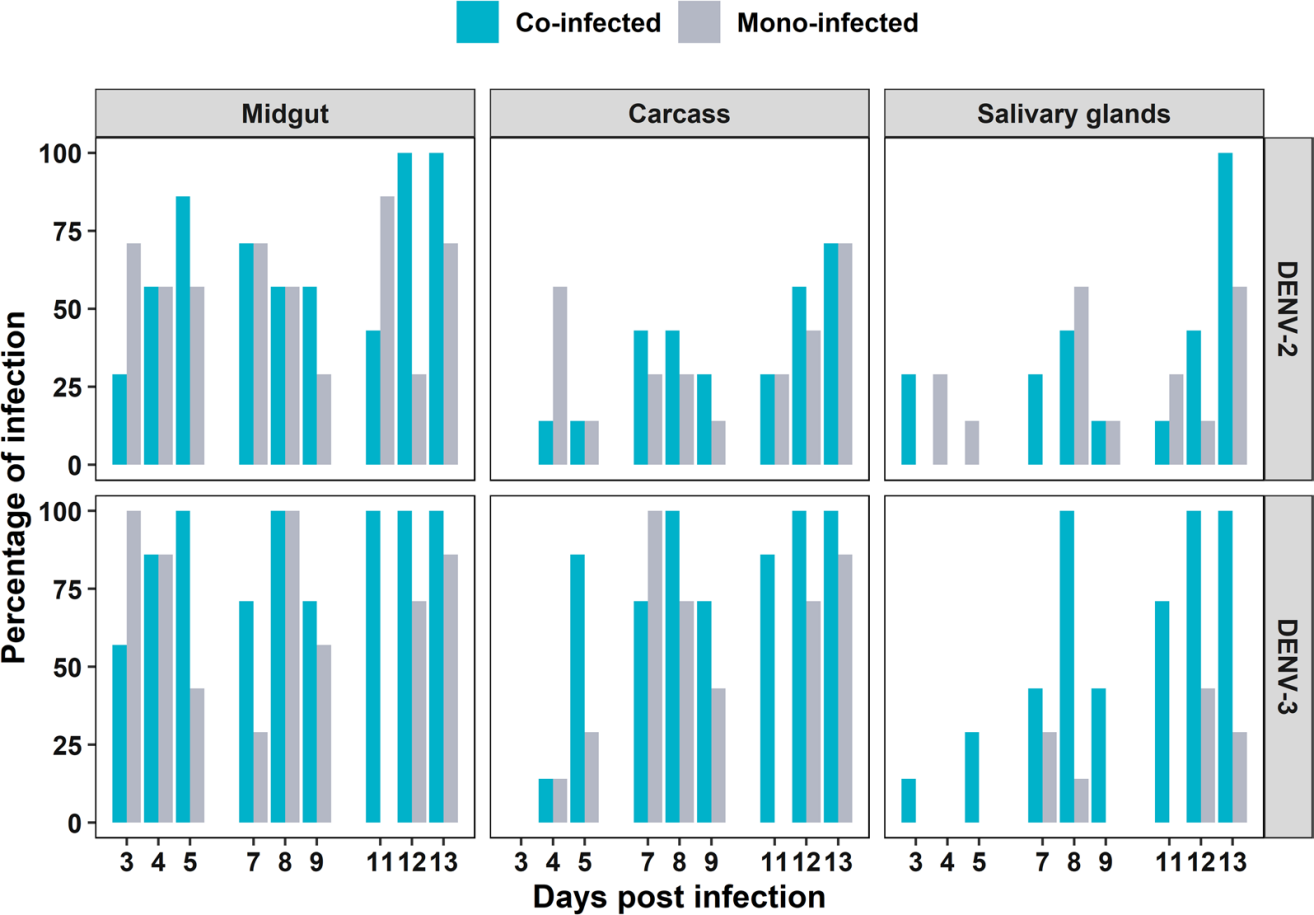
WT line susceptibility to DENV in co- and mono-infections by DPI, tissue, and serotype. *Ae. aegypti* mosquitoes were orally challenged with DENV-2 and DENV-3 in both mono- and co-infection experiments. Each bar represents the proportion of mosquitoes positive for either serotype in the mono- (grey) and co-infection (blue) experiments for each day post-infection. Mosquito tissues (midgut, carcass, and salivary glands) were collected at nine days post-infection, and DENV RNA load was determined via RT-qPCR using serotype-specific probes. Sample size *n* = 7 per day

**Fig. 5:**
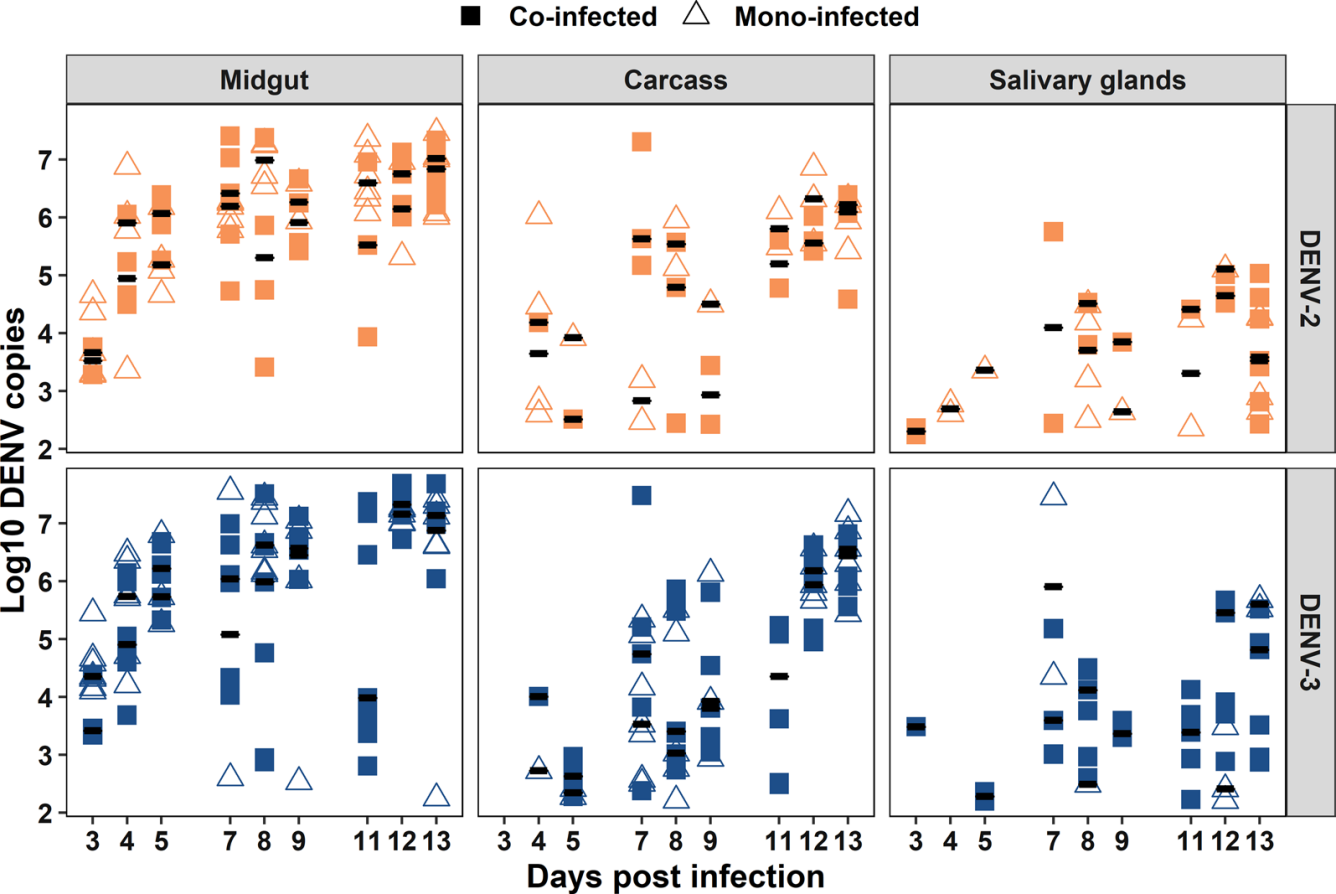
WT line DENV viral RNA load in co- and mono-infection by DPI, tissue, and serotype. *Ae. aegypti* mosquitoes were orally challenged with DENV-2 (orange) and DENV-3 (navy) in both co-infection (square) and mono-infection (triangle) experiments, with viruses offered at equivalent titers in both cases. Mosquito tissues (midgut, carcass, and salivary glands) were collected at nine days post-infection, and DENV RNA load was determined via RT-qPCR using serotype-specific probes. Mosquitoes with undetectable viral RNA load are not represented in this graph. Black bars represent treatment medians. Each symbol represents a single mosquito sample. Data were log-transformed. Sample size *n* = 7

After examining the competition dynamics between DENV-2 and DENV-3, we then sought to determine whether or not co-infection altered viral infection dynamics relative to the mono-infected state. For the *w*Mel mosquitoes, no pairwise comparisons between mono- and co-infection for each serotype were possible due to the action of *Wolbachia*-mediated viral blocking. In the WT line, we saw higher infection rates when DENV-3 was in co-infection relative to mono-infection (Fig. 4) but that the magnitude of this difference varied by DPI and tissue (df = 1, *F* = 7.5, *P* < 0.005). In the salivary glands, infection was only observed at four time points in the mono-infected state, whereas it was present in eight out of the nine time points in the co-infected mosquitoes (Fig. 4). For viral RNA load, we saw no significant difference between DENV-3 mono- and DENV-3 co-infection (Fig. 5, *χ*^2^ = 1.54, *P* = 0.21, df = 1). For DENV-2, there were no differences in either infection frequency (Fig. 4, df = 1, *F* = 0.97, *P* = 0.33) or viral RNA load (Fig. 5, *χ*^2^ = 0.01, *P* = 0.91, df = 1) between mono- and co-infection, indicating that co-infection did not alter dynamics.

### Tissue-specific differences in viral dynamics

Overall, for DENV-2, the infection rates were highest in the midgut and declined as the virus moved into the carcass and salivary glands (Fig. 4). DENV-3, in contrast, demonstrated the highest infection rates in the midgut, followed by the salivary glands (Fig. 4). Viral RNA loads for the two serotypes were highest in the midgut and decreased in the carcass and then the salivary glands (Fig. 5, DENV-2; *χ*^2^ = 57.48, *P* < 0.001, df = 2; DENV-3; *χ*^2^ = 57.48, *P* < 0.001, df = 2). The carcass was the tissue most likely to have either serotype present despite the action of *w*Mel-mediated pathogen blocking. Dissemination rates of each of the two serotypes in the presence of *w*Mel were similar across all three tissues (Fig. 1).

## Discussion

We sought to explore the role of DENV serotype co-infection and competition in both *Wolbachia*-mediated blocking and within-vector infection dynamics. In particular, we examined DENV RNA loads and infection rates in DENV-2/DENV-3 co-infected *Ae. aegypti* midguts, carcasses, and salivary glands of two mosquito lines with (*w*Mel) and without *Wolbachia* infection (WT). We identified that in WT mosquitoes, there was a competitive advantage for DENV-3 when co-infected with DENV-2 across multiple tissues compared to a mono-infection. Additionally, we saw a strong pathogen-blocking phenotype in *w*Mel mosquitoes independent of co-infection status, tissue, and DPI.

Our data showed that for the WT mosquitoes, DENV serotype co-infection altered the infection frequency of each serotype in a tissue-specific manner, with DENV-3 having a competitive advantage over DENV-2. This advantage was clearer at the dissemination stage of infection once the virus reached the hemocoel from the midgut and ultimately at the transmission level when the virus arrived at the salivary glands. Moreover, the DENV-3 competitive advantage was confirmed when we compared each serotype in co-infected mosquitoes vs mono-infected mosquitoes, and we identified that DENV-3 produced higher infection rates in all tissues when mosquitoes were co-infected. Conversely, DENV-2 infection dynamics did not change significantly when mosquitoes were co-infected compared to mono-infected. Only one other study has looked at the infection dynamics of co-infection with DENV-2 and DENV-3 both in vitro and in vivo [20]; contrary to our findings, they showed an increase in replication efficiency of 1000-fold for DENV-2. These contradictory results may be due to the use of different DENV and mosquito genotypes.

When the replication capacities of the two DENV serotypes were assessed in WT mosquitoes, no significant differences in viral RNA load between DENV-2 and DENV-3 in either mono- and co-infection were found. Differences in viral replication rates without affecting the viral RNA load in each tissue have been previously reported [14], in which DENV serotypes that had high growth rates did not necessarily achieve high viral RNA loads or high infection frequencies in experimental mosquito populations. The disconnect between viral RNA load and infection frequency for each virus suggests that stochastic processes are potentially taking place or/and genotype-by-genotype interactions are affecting the virus infection dynamics. For example, previous research has shown that the strength of the mosquito immune response can be tissue- and serotype-dependent [34–36] and could lead to scenarios in which mosquitoes are more susceptible to dengue infection with a particular serotype (i.e., higher infection frequency) but can also mount a relatively strong immune response in particular tissues (i.e., low viral RNA load).

Arboviral co-infection is not limited to DENV serotypes; although most reported cases are with DENV, co-circulation of DENV with CHIKV and/or ZIKV is increasing around the globe [37, 38]. This co-circulation represents a major challenge for many national and international public health organizations, particularly because there is little information about the potential clinical and biological consequences of these interactions. Individual case reports of arboviral co-infection in humans suggest enhanced disease severity. Co-infection with ZIKV and CHIKV has been associated with severe meningoencephalitis in a male patient, and co-infection with DENV and CHIKV was linked to severe metrorrhagia in a female patient [39, 40]. Increased disease severity may occur when both viruses interfere with different parts of the same immune pathways. For example, interferon signaling is a major part of the human antiviral response, and it is mediated by the signal transducer and activator of transcription one and two (STAT-1 and STAT-2) [41]. DENV has been shown to block STAT-1, and CHIKV can potentially interfere with STAT-2 [42], therefore blocking the activation cascade of interferon and potentially increasing disease severity.

The *w*Mel mosquitoes challenged in DENV serotype co-infection were far less susceptible than the WT line, indicating that the pathogen-blocking phenotype caused by *Wolbachia* infection is not affected by concomitant DENV serotypes. Additionally, the effect of *Wolbachia* blocking was seen in all three tissues and was stable across nine time points, from 3 to 13 DPI, encompassing days of the mosquito’s lifespan relevant to viral transmission in the field. Pathogen blocking by *Wolbachia-*infected mosquitoes challenged with co-infecting arboviruses has only been shown once before [43]. Co-infection was performed using DENV/ZIKV challenges but not with multiple DENV serotypes at the same time, and it was limited to three time points and only one mosquito tissue. Although co-circulation of novel emerging arboviruses like ZIKV or CHIKV coupled with DENV has been reported [44–46], most countries where DENV is endemic have reported co-circulation of all four DENV serotypes, resulting in hyper-endemicity for the virus [47, 48]. Specifically, for DENV-2 and DENV-3 infections, one study showed that from 303 human serum samples, up to 21% were infected with both viruses [49]. This phenomenon has been linked to an increased frequency of severe dengue cases and an overall increase of virulence [50].

Another effect of co-transmission can be epidemiological, where co-infection occurring from a single biting event can significantly increase disease burden. Mathematical modeling using in silico data in *Ae. aegypti* has shown that co-transmission events can potentially lead to an increased number of cases for both viruses [17]. An additional unanswered question in co-infection in *Ae. aegypti* is how sequential viral infections might affect pathogen transmission dynamics, a scenario that can also occur in nature (51, 52). Whether or not viruses can potentially interact after sequential acquisition by the mosquito has yet to be determined. Last, in our co-infection experiments, we used half as much of each virus but the same overall viral RNA load as mono-infections; however, no statistically significant differences were observed in viral RNA load between mono-infection and co-infection for either serotype. This still begs the question of the potential outcome of infecting the mosquitoes with twice the amount of each serotype and twice the overall amount of virus in co-infection.

## Conclusions

Here, we present the first examination of DENV serotype co-infection and its effect on *Wolbachia*-mediated pathogen blocking. We demonstrated that the *w*Mel mosquito strain is capable of blocking DENV serotype co-infection in a systemic way across the mosquito body. Moreover, we showed that for WT mosquitoes, serotype co-infection can affect infection frequency in a tissue- and time-specific manner and that both viruses have the potential of being transmitted simultaneously.

## Supplementary Information

**Additional file 1**: **Figure S1**. Relationship between PFU and viral RNA copies for both DENV-2 and DENV-3. Viruses were harvested from C6/36 cells at DPI 5 and 7 and on the day of the experiment (DPI7-Exp). A) PFU/ml for both DENV-2 and DENV-3 for DPI5 (red), DPI7 (blue), and on the day of the experiment DPI7 (black). B) Log10 viral RNA copies/ml for both DENV-2 and DENV-3 for DPI5 (red), DPI7 (blue), and on the day of the experiment DPI7 (black). All assays were done using live virus

## Data Availability

All data for this study can be found at Figshare https://doi.org/10.6084/m9.figshare.14050352.
