# Supplementary material for: The effects of DENV serotype competition and co-infection on viral kinetics in Wolbachia-infected and uninfected Aedes aegypti mosquitoes
Source: Parasit Vectors. 2021 Jun 9;14:314. doi: 10.1186/s13071-021-04816-0 (PMC8190863; doi:10.1186/s13071-021-04816-0)
Supplement: Supplementary file 1 — Additional file 1: Figure S1. Relationship between PFU and viral RNA copies for both DENV-2 and DENV-3. Viruses were harvested from C6/36 cells at DPI 5 and 7 and on the day of the experiment (DPI7-Exp). A) PFU/ml for both DENV-2 and DENV-3 for DPI5 (red), DPI7 (blue), and on the day of the experiment DPI7 (black). B) Log10 viral RNA copies/ml for both DENV-2 and DENV-3 for DPI5 (red), DPI7 (blue), and on the day of the experiment DPI7 (black). All assays were done using live virus [file 13071_2021_4816_MOESM1_ESM.docx]

**
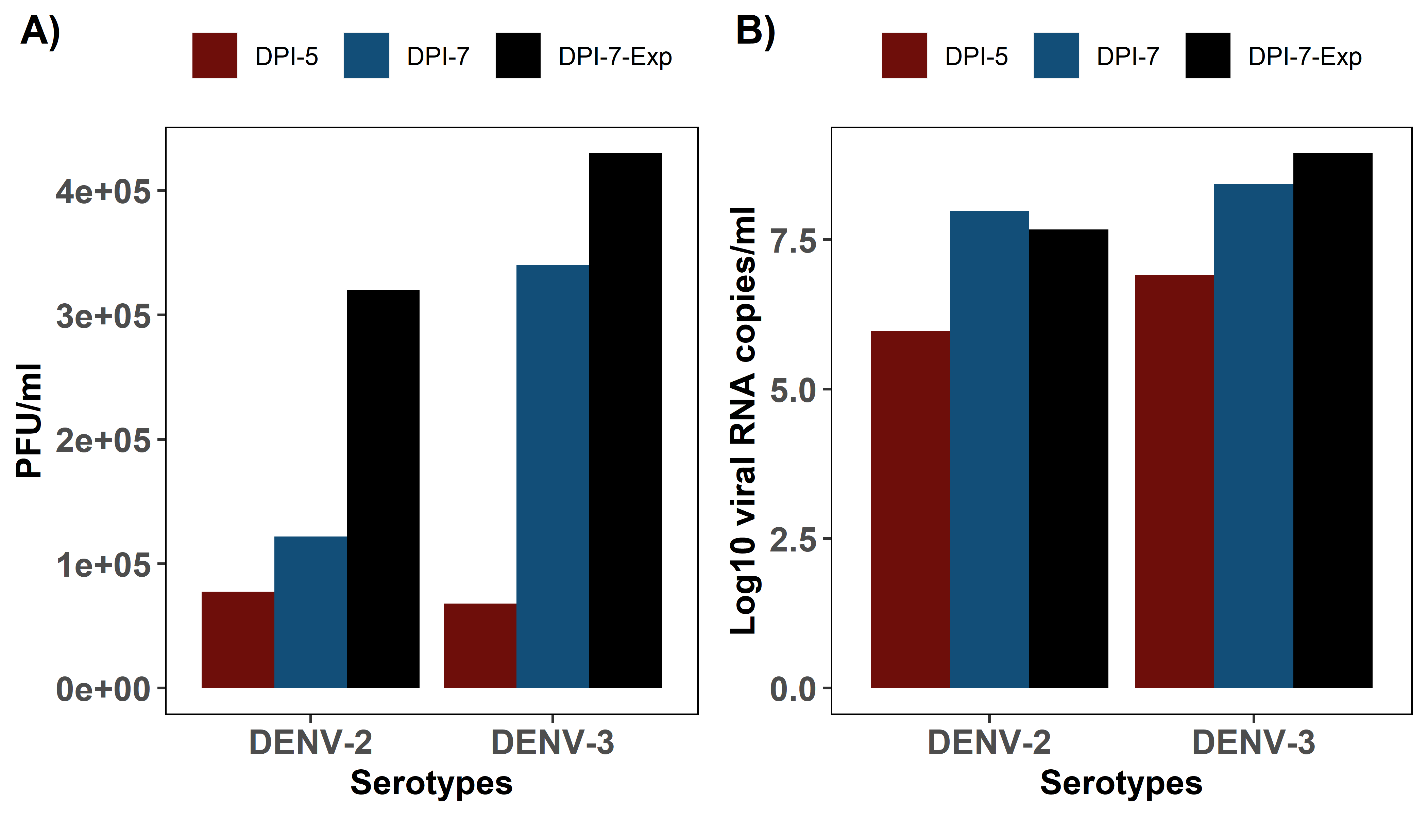
Supplementary figure 1. Relationship between PFU and viral RNA copies for both DENV-2 and DENV-3**. Viruses were harvested from C6/36 cells at DPI 5 and 7 and on the day of the experiment (DPI7-Exp). **A)** PFU/ml for both DENV-2 and DENV-3 for DPI5 (red), DPI7 (blue), and on the day of the experiment DPI7 (black). **B)** Log10 viral RNA copies/ml for both DENV-2 and DENV-3 for DPI5 (red), DPI7 (blue), and on the day of the experiment DPI7 (black). All assays were done using live virus.
